# Robustness of open source community multi-project knowledge collaboration network based on structural hole theory

**DOI:** 10.1371/journal.pone.0292444

**Published:** 2024-01-02

**Authors:** Shaojuan Lei, Chenzhi Wang, Taoge Zhang, Xinhua Liu

**Affiliations:** Nuclear and Radiation Safety Center, MEP, Beijing, China; LUMSA: Libera Universita Maria Santissima Assunta, ITALY

## Abstract

Nodes in the structural hole position play a key role in the multi-project network of the open source community (OSC). This paper studies the robustness of this network based on structural hole theory. First, a semantic-based multi-project KCN is constructed, and four node types are identified: knowledge contribution nodes, knowledge dissemination nodes, structural hole nodes (SHNs) and opinion leader nodes. Second, a robustness analysis model of the edge failures of these four key nodes is constructed. Third, a simulation test is conducted on the proposed model using empirical data from the Local Motors multi-project OSC. The results show that the KCN has the lowest robustness when facing the edge failure of opinion leader nodes, followed by knowledge dissemination nodes, knowledge contribution nodes, SHNs and random nodes. The edge failure of opinion leader nodes causes the lowest network robustness because of the propagation effect of these nodes. Additionally, SHN failure has only a small initial impact on connectivity, whereas knowledge collaboration efficiency decreases rapidly (i.e., the edge failure of SHNs causes the network to enter a state of high connectivity and low efficiency). The proposed model can be used to provide comprehensive and targeted management guidance for OSC development.

## Section 1 Introduction

The concepts of "open source" and "free software" emerged in the 1980’s, followed by open source communities (OSCs) such as Apache, Debian, Linux and Mozilla, which are still enjoying worldwide success. The open source model provides advantages that traditional production methods cannot match (i.e., low cost and high collaborative innovation), and it has infiltrated all fields of social development from the software industry which obtain many successful applications [1, 2]. Scholars have carried out extensive analysis and research on the OSC and its representative open source economy. As an innovative design model whose core characteristics are the self-organization process and large-scale, deep collaboration of its users, its most critical elements are undoubtedly "people" and "collaborative behavior". Specifically, volunteers from all over the world gather in OSCs to share ideas and knowledge, and to design and produce products and services with open source advantages [3, 4]. Therefore, the promotion of collaboration quality and the number of users are important guarantees for community development. Li et al. [5] stated that the autonomy and mobility of community participants have an important impact on the network structure and sustainable development of the community, which affects the success or failure of open source design. Crowston et al. [6] stated that the quality and quantity of user participation and collaboration are more important than the number of volunteers entering the community. Griffith [7] and Singh [8] both suggested that community members’ reliance on their own experiences and knowledge when collaborating with other members is important to the completion of knowledge sharing and the improvement of development efficiency. Ransbottham et al. [9] stated that a primary reason for the inefficient operation of open source projects is the lack of truly valuable contributions by community members.

Some studies also suggested that the OSC built by most Fortune 100 companies did not achieve the expected results and returns in terms of product innovation and economic benefits because of low user collaboration, lack of valuable contributions, serious loss of user resources and other factors [10, 11]. This is one demonstration that behind many successful open source design cases, there are also many failed projects and communities.

By comprehensively analyzing the influencing factors in the open source design process, this paper builds a network model that fully reflects the characteristics of the OSC. This includes using topological information to identify the key nodes with different characteristics in the network and analyzing network robustness when collaborative behavior fails. The model can reveal effective guidance for the stable and efficient development of the OSC and the construction of a perfect, open source, ecological environment.

## Section 2 Related work

### Identification of key nodes

The identification of key nodes and their impact on network structure and performance has been a primary focus of complex network research [12]. The core principle of the social network analysis method is "importance is equivalent to conspicuousness" [13]. Mining important nodes involves not destroying the integrity of the network. Generally, the importance of a node can be measured using centrality indicators (e.g., degree centrality, betweenness centrality, proximity centrality and the structural hole position), which describe the importance of a single node in the network from different angles [14]. For example, Bonacich et al. [15] first proposed the use of the degree value to show the importance of nodes: the greater the node degree, the stronger its importance in the network. Zhu et al. [16] combined network centrality and user activity to build a key node index system, then used the grey correlation model to rank key nodes to identify the opinion leader nodes in the network. Many studies show that key node ranking results obtained through a comprehensive use of measurement indicators are better than those obtained through use of a single indicator [17, 18]. However, few studies consider the key node sequencing problem for structural holes using current synthesis methods. Structural hole nodes (SHNs) have unique characteristics that distinguish them from core nodes in the complex network, so they are often easily ignored even if they play an important role in the network. Additionally, research on structural holes in complex networks is not extensive enough, and it is still worth exploring how to scientifically measure structural holes in this setting [19].

In their research on the OSC multi-project complex network, Zhou [20, 21] and Xu [22] included the structural hole position when identifying key network using the comprehensive sorting method. However, their network constructions only considered the collaboration frequency, not the collaboration content, which is unrealistic. Lei et al. [23] conducted research on the dynamic robustness of the OSC knowledge collaboration network (KCN) based on opinion leader identification, ranking nodes by degree centrality, betweenness centrality and TOPSIS multi-attribute decision-making methods. They considered the structural hole position in the identification of opinion leader nodes, but their research was limited to a single project network, giving it a certain one-sidedness. In the OSC, SHNs that connect multiple projects or groups are more important than SHNs that are part of only one project or group, as the removal of multi-project SHNs significantly increases the node distance between groups [24]. To fill these research gaps, this paper fully considers the structural hole position when identifying the key nodes of the multi-project network, and both collaboration frequency and collaboration content are considered in the constructed KCN.

### Identification and utility of SHNs

When no direct connection exists between two or more individuals in the network, it creates a structural vulnerability, which widely exists in many types of networks (e.g., social, innovation, knowledge, collaborative and information networks) [25]. The development of structural hole theory remains driven by the need to understand how entities benefit from the competition of social networks and their cross relationships. Structural hole theory is often used to analyze the relationships between individuals, organizations or other entities occupying social networks. Individuals in the structural hole position can (1) effectively fill the gap between two groups with complementary resources and knowledge, (2) bring new and valuable knowledge to the two groups, and (3) bring competitive and innovative advantages to the network.

There are two measurement systems for structural holes: Burt’s structural hole index, which is suitable for measuring the structural holes of individual networks; and Freeman’s betweenness centrality index, which is applicable to the whole network [26]. Burt’s structural hole theory has been used by many scholars in the fields of commercial economics [27], computer science [28], libraries and information [29], public management [30] and social psychology [31], making many valuable achievements.

Research shows that individuals or organizations in the structural hole position have natural location advantages [32]. For example, Kim et al. [33] investigated the position of influential Twitter users in the social network, finding that information was disseminated more widely when it was created by users in the structural hole position. Further, Zhou et al. [34] stated that individuals in the structural hole position can access non-redundant knowledge, bringing competitive advantages that mainly come from their access to (and control of) information between different groups.

Structural holes can also play an important role in innovation. For example, Wang et al. [35] found that the structural holes of collaborative networks promote exploratory innovation, while point-degree centrality has the opposite effect. Further, Stea and Pedersen [36] examined the impact of structural holes on innovation in different working environments, stating that the organizational environment is a network environment, and social interaction is an important driving force for employee innovation.

Structural holes can also impact node behavior. For example, Figueiredo et al. [37] investigated the influence of network centrality, structural holes and connection strength on the recipient’s behavior of selecting content, finding that SHNs can effectively express the recipient’s behavior when selecting content. Further, Wang et al. [38] found that interaction experience is the direct factor to promote customer innovation behavior when discussing customer innovation behavior in brand community. Customers with high centrality and structural holes can produce more customer innovation behavior after experimenting with better interaction experience. At present, theoretical research on structural holes mainly focuses on the identification, embedding and utility of structural holes [39]. There are relatively few research examples and achievements based on the OSC, particularly regarding the impact of structural hole node failure (or collaborative behavior failure) on multi-project network performance.

### Research on the robustness of OSC network

The OSC network is an effective expression of user participation behavior, the user collaboration process and the dynamic evolution process of the community in the operation of OSC using complex network theory and social network analysis method [40, 41]. The construction of an OSC network generally involves using developers as nodes, and users’ common project experience or the interaction experience between users as edges. They are mostly undirected and unweighted networks. Only a small number of networks are weighted networks that consider the frequency of collaboration between users. For various types of OSCs (e.g., product communities, design communities, content communities), the strength of the links between nodes is key to measuring whether the network structure accurately reflects the community structure [42, 43].

OSC network robustness refers to the ability of the system to maintain structural integrity and functional continuity when the network is disturbed or damaged [44, 45]. Robustness analysis can effectively identify the core elements of community development. In addition, it can identify the direction for system management and risk control, to create a resilient and efficient community ecology. Research on the robustness of complex networks began in 2000 when Albert et al. [46] investigated the impact of topology on the network. The robustness of OSC networks has also gradually become more of a research focus. For example, Mark Fuge et al. [47] found that the OSC design network has a core-edge structure, making it naturally robust to attacks. Further, Zhang et al. [48] studied the topological characteristics and robustness characteristics of the Open IDEO network from different time spans of community development, finding that the network’s robustness performance and network protection focus are different at various stages of development. Zhou et al. [20, 49] studied the impact of user loss on the robustness of open source projects and OSCs, finding that the community is more sensitive to the loss of key users, with a continuous loss of key users causing the rapid collapse of the network. In their study of multi-project networks, it is also found that the loss of opinion leader nodes causes the “following” behavior of surrounding nodes (i.e., the failure propagation among the surrounding nodes).

In their research on the robustness of open source projects, Lei et al. [50, 51] divided edges into contribution behavior and propagation behavior according to their topological characteristics. They then assessed the impact of collective edge failure and successive edge failure on network performance, finding that failure propagation causes more serious damage to the network. They further studied the network robustness of the OSC under the failure of the collaborative behavior of different nodes, finding that the failure of opinion leader nodes leads to the failure of other nodes, which causes the rapid collapse of the network. However, none of the above studies considered the multi-project OSC network. In subsequent research, Zhang [52] divided nodes based on their structural characteristics and project characteristics; they then investigated the robustness of the multi-project OSC network under node failure and edge failure, but they did not consider the impact of node failure at the structural hole position or its edge failure on the network when dividing nodes.

As shown above, most existing research on the robustness of OSC networks does not consider the behavioral characteristics of SHNs when constructing attack strategies or failure modes. Further, when using the comprehensive analysis method to identify the key nodes, the structural hole position is rarely considered. In the multi-project network, SHNs can connect different groups or node organizations of different projects, and they play a key role in community development and project completion. Therefore, it is pertinent to consider the structural hole position in key node identification and robustness research. Further, most existing studies carried out comprehensive research on network robustness during node failure, but the research on node behavior failure is relatively small. Therefore, it is necessary to design specific failure modes for nodes with different characteristics, to research the impact of their behavior failure on the network.

In summary, based on the characteristics of multi-project knowledge collaboration in the OSC, this paper (1) constructs a directed, weighted, semantic-based multi-project KCN by combining two key factors among project contributors: comment content and comment frequency. (2) Network nodes are categorized into knowledge contribution nodes, knowledge dissemination nodes and SHNs based on three single indicators, and the comprehensive analysis method is used to identify opinion leader nodes by integrating four centrality indicators (i.e., strength centrality, betweenness centrality, proximity centrality and the structural hole position). (3) Four edge failure modes are designed based on the above node types. Regarding the opinion leader node failure mode, the SIR model of infectious disease is applied to construct the influence propagation model. (4) Using the empirical data of multiple projects in the Local Motors open source automobile design community, a robustness simulation model is built to simulate the change trend of network performance under the four edge failure modes. The subsequent simulation test results provide a basis for the formulation of community management strategies.

## Section 3 Methods

### Construction of multi-project KCN and identification of key nodes

#### Materials

This paper uses the Local Motors OSC as a typical research object. In addition to its avant-garde design concept of "producing for customers", this community also produces customized automobile products that have attractive, high-tech components.

Users of the Local Motors OSC can freely choose the design projects they are interested in, exchange creative ideas and put forward suggestions for improvement. The design scheme can be cycled forward in this collaboration, tested, improved, and retested, and then can be jointly manufactured with customers who buy products after finalization.The following are the specific steps for data collection and processing:

1. Data acquisition: The research team used Python web crawler framework tools (Scrape and PySpider) to obtain raw data. we select 11 projects from May 2008 to November 2016: 3D-PC, Airbus C, Air C, Darpa, LM SF-01, Olli, Open T, Rally F, Road R, Sketchover and Verrado DT. We crawl the complete collaboration data from which includes project information, time information and comments.
2. Data cleaning: Data cleaning ensures the integrity and accuracy of data, and we carry out data cleaning in three steps.(a) De duplication: Delete or merge duplicate records in the data. (b) Missing value processing: Complete missing information by interpolation or filling in gaps. (c) Abnormal value handling: Operations such as review, correction, or deletion are required to handle data that does not conform to the format.
3. Data filtering: Data filtering removes identification data from comment information and preserves research content. Identification data includes information such as name, initials, physical address, Internet Protocol (IP) address, etc. After filtering the data, item numbers, user numbers, user comment relationships, comment content, comment times and other content are kept. Through statistics analysis, the total number of users for knowledge collaboration is 1410, and the total number of collaboration times is 18469.

Statement: The raw data for this study comes from the open source community: https://Localmotors.com. The data collection method complies with the terms and conditions of the website. Part of the original data can be viewed in the S1–S11 Data.

#### Construction of multi-project KCN

**Fig 1:**
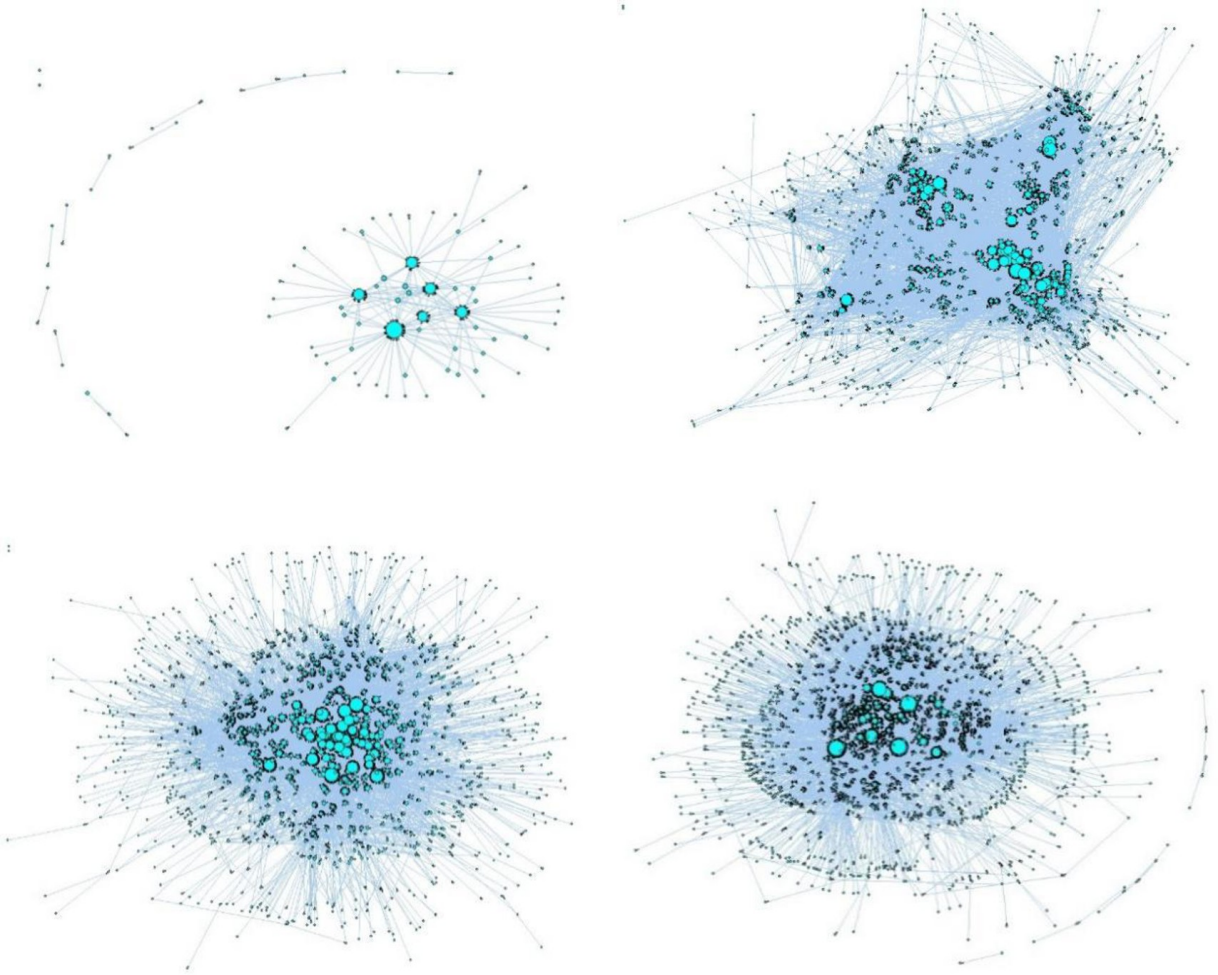
Evolution diagram of semantic-based multi-project knowledge collaboration network (KCN).

To better simulate the knowledge collaboration behavior among community members, a semantic-based multi-project KCN is established. Users participating in knowledge collaboration are represented by node set **V** and collaboration relationship is represented by relationship set **E**. To more accurately express the depth and intensity of collaboration between users, we use the weighted processing of collaboration content and collaboration frequency as the edge weight, which is represented by the edge weight set ***W***. In summary, Fig ***G*** = (***V***, ***E***, ***W***) is the multi-project KCN model. The construction method of the semantic-based multi-project KCN and the calculation process of the edge weight are both detailed in previous research by Lei [50, 51]. Based on the calculation results, the network evolution diagram is drawn using UCINET6.4, as shown in Fig 1.

In Fig 1: the dots represent users in the network; the larger a node’s point weight, the larger its area; the connecting edge indicates the connection between users, and the arrow indicates the direction of collaboration. Fig 1 shows that as time passes, more users enter the community for knowledge collaboration. Users who participate in different project collaboration form a cluster. With the completion of some existing projects and the start of new projects, users collaborate in multiple projects, resulting in the connection of many nodes. There are almost no free nodes, which may be because the processing of the original data involves the removal of all behaviors that do not participate in knowledge collaboration.

The closer a node is to the core of the network, the larger the area and the more frequent the collaboration with other nodes, reflecting the obvious "core edge" structure. The scale, some static topology parameters and network characteristics of the semantic-based multi-project KCN are measured using UCINET software, as shown in Table 1.

**Table 1:** Network topology parameters and structural characteristics of the multi-project KCN.

Network Size		Topological Parameters				Structural Characteristics		
Nodes	Edges	Average out-strength	Density	Average path length	Clustering coefficient	Small-world characteristic	Scale-free property	Assortativity
1410	18469	0.119	0.0001	3.105	0.097	Yes	Yes	No

Note: According to Davis, Yoo and Baker [53], the small-world parameters can be expressed as SW = [Cactual/Lactual]*[Lrandom/Crandom], where Cactual is the average clustering coefficient of the network, Lactual is the average path length, Lrandom=ln(n)/ln(‹k›),Crandom=‹k›/n,n is the number of nodes and ‹k› is the average degree. The network efficiency is $E=\frac{1}{n(n-1)}\sum_{i\neq j}\frac{1}{d_{ij}},where\;d_{ij}$ is length of the weighted shortest path from node i to j [54].

The evolution process of the network and Table 1 demonstrate that the frequency of knowledge collaboration is relatively high, and the vast majority of users entering the community have a strong sense of participation. Most users still have great collaboration potential, so it is pertinent to analyze the knowledge collaboration behavior of different users and give corresponding incentive strategies. Further, network users form multiple communities of different sizes, and the SHNs between each community increase the connection and knowledge collaboration of the whole network. Therefore, it is necessary to take the structural hole position as an essential indicator when identifying the key nodes of the multi-project KCN. Finally, the community aggregation coefficient is large and the average path is short. This indicates that the network has obvious small-world characteristics.

Ahmad et al [55]. found that network structures with small world characteristics play an important role in the dynamic propagation of behavior. Many scholars have also verified the process of behavior propagation in small world networks through research in fields such as knowledge dissemination [56], virus dissemination [57], rumor dissemination [58], and behavior dissemination [59]. On the theory of "two-level communication" and "opinion leaders", Lazarsfield [60] and Katz [61] believe that opinion leaders are usually active elements in the process of mass communication, and information communication is carried out according to the model of "media opinion leaders audience". Therefore, in multi-project KCN, it is also necessary to conduct research on opinion leaders and their propagation behavior.

### Structural hole theory and calculation results

In 1992, Burt [62] introduced structural hole theory, studied the structural form of human networks, and analyzed what kind of network structure can bring more benefit or reward to network action subjects. Structural holes refer to non-redundant connections in complex networks. Structural hole theory has been widely applied in the field of social science. Research on social networks mainly focuses on two aspects: (1) exploring the embedding and utility of structural holes in social networks, and (2) identifying the key subjects of information dissemination (i.e., opinion leaders) [63]. Burt’s structural hole measurement index considers four main aspects (i.e., effective size, efficiency, constraint and hierarchy), as detailed in Table 2.

**Table 2:** Measurement index and interpretation of structural hole.

Structural hole index	Calculation formula	Interpretation	Relevance
effective size	${ES}_{i}=\sum_{j}\left(1-\sum_{q}p_{iq}m_{jq}\right)q\neq i,j$ (1)	Measures the overall influence of a node. Refers to the effective scale of an actor and the individual network size of the actor minus the redundancy of the network (i.e., the effective scale equals the non-redundant factor of the network).	Positive correlation: the larger the effective scale of actors, the greater the possibility of having structural holes.
efficiency	EI=ESiSi,q≠i,j (2)	Describes the impact of nodes on other nodes in the network. The efficiency of nodes at the location of structural holes is generally high.	Positive correlation: the higher the efficiency, the more efficient the node’s action in the network, and the faster the information dissemination.
constraint	$C_{ij}={\left(p_{ij}+\sum_{q}p_{iq}p_{qj}\right)}^{2},\;q\neq i,j$ (3)	Refers to the limit degree of the ability of the node to use structural holes in its own network. The degree of dependence of nodes on other nodes is taken as the evaluation criteria.	Negative correlation: the higher the constraint coefficient, the closer the network where the node is located, and the fewer the number of structural holes.
hierarchy	$H=\frac{\sum_{j}\frac{C_{ij}}{C/N}\ln\frac{C_{ij}}{C/N}}{N\;\ln\;N}$ (4)	Refers to the degree of restrictive concentration on one actor.	Negative correlation: the lower the hierarchy, the more core the node is.

Note: In Eq (1), *j* represents all nodes connected to node *i*, q is a third party other than *i* or *j*, *p*_*iq*_*m*_*jq*_ represents the redundancy between node *i* and node *j*, and *m*_*jq*_ refers to the marginal strength of the relationship between *j* and *q*; In Eq (2), *S*_*i*_ represents the actual scale of the individual network of node *i*, and *p*_*ij*_ represents the proportion of the relationship between node *i* and node *j*; In Eq (3), *p*_*ij*_ represents the direct relationship between *i* and *j*, and ∑_*q*_*p*_*iq*_*p*_*qj*_ represents the indirect relationship between *i* and *j*; In Eq (4), *N* is the actual scale of the individual network.

The above four indicators are used to analyze the structural hole characteristics of KCN. Table 3 shows the structural hole index values of the top ten nodes on the multi-project KCN, ranked according to the constraint indicator. Four of these top ten nodes have high betweenness values and point strength values (among the top 10 nodes), which further demonstrates the structural attributes of SHNs. The SHN is the only channel connecting two groups or two nodes in the network. It has the absolute location advantage in the community, but it is not necessarily the node with the most collaboration or the most critical location. Therefore, consideration of the structural hole is necessary both in network key node identification and network robustness research.

**Table 3:** Top ten nodes based on constraint coefficient.

node	constraint	effective size	efficiency	hierarchy
52086	0.046	92.374	0.983	0.276
52	0.076	281.087	0.887	0.629
1347	0.08	234.077	0.88	0.605
1439	0.08	301.172	0.932	0.607
407	0.084	171.11	0.873	0.537
907	0.09	110.346	0.89	0.404
10	0.095	126.439	0.897	0.488
1731	0.097	71.066	0.877	0.305
12640	0.107	51.976	0.945	0.323
205	0.109	10.2175	0.851	0.544

#### Multi-project network opinion leader identification and influence model

*Identification of opinion leaders*. In social networks, the key nodes that comprehensively consider the structural and topological attributes of nodes are usually called opinion leaders. Identification results based on multiple indicators are more comprehensive and accurate than those based on only a single indicator. In their research on the identification and “following” effect of opinion leader nodes in the OSC, Xu [22] found that–similar to in other forms of networks (e.g., social networks, software networks and business networks)–opinion leader nodes dominate knowledge sharing, information dissemination, public opinion orientation, behavior and decision-making guidance. Based on the constructed directed, weighted, semantic-based multi-project KCN, four indexes are calculated using UCINET analysis software and SPSSAU software: node strength centrality (SC), closeness centrality (CC), betweenness centrality (BC), and the structural hole position (SH). The entropy weight TOPSIS multi-attribute decision-making method is proposed to identify opinion leaders. First, the four indicators are standardized using the decision matrix shown in Table 4. The information entropies of the four indicators are 0.9031 for SC, 0.9387 for CC, 0.9076 for BC and 0.9442 for SH, and the respective weights are ***w***_***SC***_ = **0.3163**, ***w***_***CC***_ = **0.20**, ***w***_***BC***_ = **0.3016** and ***w***_***SH***_ = **0.1821**.

**Table 4:** Four index standardization matrix.

Support Surface	SC	CC	BC	SH
1	0.0807	0.8614	0.0785	0.27
2	0.0234	0	0	0.643
3	0.0138	0	0	0.556
4	0.091	0.8614	0.0542	0.236
5	0.0246	0.8614	0.0298	0.382
…	…	…	…	…

The weighted standard decision matrix, Y, is:

$$Y=[\begin{array}{cccc} 0.0253 & 0.1723 & 0.0237 & 0.0491\\ 0.0073 & 0 & 0 & 0.1170\\ 0.0043 & 0 & 0 & 0.1012\\ 0.0285 & 0.1723 & 0.0163 & 0.0429\\ 0.0077 & 0 & 0.0089 & 0.0695\\ 0.0250 & 0.1723 & 0 & 0.1243\\ 0.0895 & 0.1723 & 0.0778 & 0.0931\\ 0.0032 & 0 & 0.0131 & 0.1307\\ \cdots & \cdots & \cdots & \cdots \end{array}]$$

Next, the positive and negative ideal scheme is generated:

$$A^{+}=(0.319,0.202,0.305,0.184)$$

$$A^{-}=(0.003,0.002,0.003,0.002)$$

**Fig 2:**
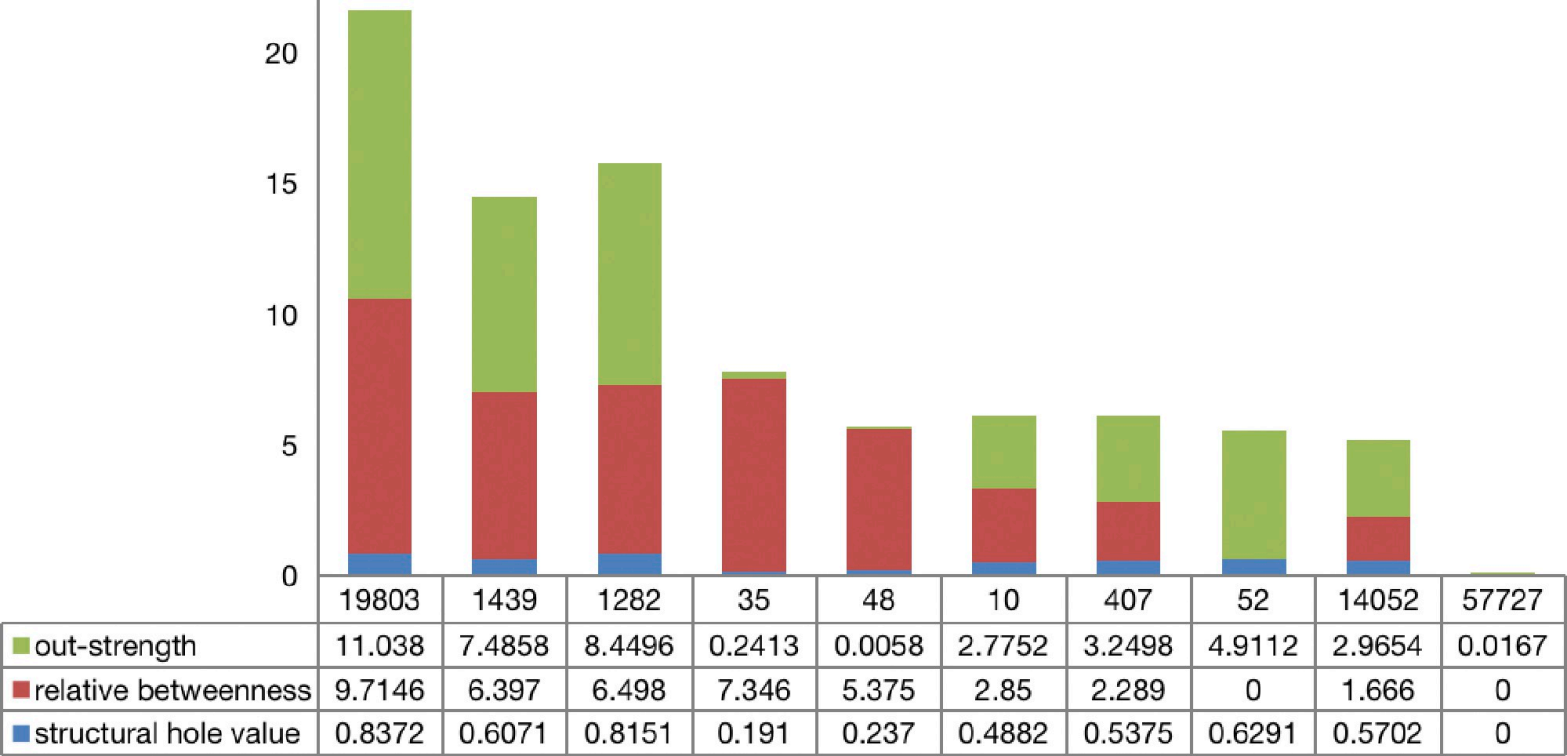
Out-strength, relative betweenness and structural hole values of opinion leader nodes.

The distance **D** and closeness **Z** from each decision scheme to the ideal scheme are calculated. The specific results are shown in Table 5, sorted according to closeness **Z**. Fig 2 compares the out-strength, relative betweenness and structural hole values of the top ten nodes. Here, eight nodes coincide with the top ten nodes in terms of out-strength value and seven nodes coincide with the top ten nodes in terms of betweenness value. This shows that users with professional knowledge, active collaboration with others and "intermediary" status have greater influence in the community. This conclusion has also been verified in the identification of opinion leaders in the single-project KCN [23]. Additionally, among the top ten nodes, there are also nodes with low out-strength values and betweenness values.

**Table 5:** Opinion leader identification results.

Node	$D_{i}^{+}$	$D_{i}^{-}$	Z_i_
19803	0.155	0.471	0.752
1439	0.185	0.353	0.656
1282	0.2	0.372	0.65
35	0.238	0.303	0.559
48	0.335	0.303	0.474
10	0.356	0.266	0.428
407	0.331	0.229	0.409
52	0.334	0.228	0.405
14052	0.36	0.245	0.405
57727	0.354	0.217	0.38

*Behavior influence model of opinion leaders*. In the multi-project KCN, the influence model of opinion leader behavior refers to the model design detailed in during former research on the single-project network [23]. The opinion leader nodes in the multi-project network are taken as the infectives, ***I***. After the collaboration behavior failure of the opinion leader, this failure is transmitted to other susceptibles, ***S***, with infection probability ***λ***. The infected node also recovers the willingness to collaborate with recovery probability ***γ*** and becomes the removal, ***R***. Because the infection probability changes dynamically with the extension of the infection path, the infection probability ***λ*** is represented by a negative exponential model, $f(t)=e^{-\mu (t-t_{0})}$, where the characteristic scaling factor, ***μ***, is represented by the reciprocal of the standard deviation coefficient.

The standard deviation coefficient is a relative index reflecting the degree of dispersion of vertex strength, so we use its reciprocal to express ***μ***. In the directed weighted network, 〈***ω***〉 is the average weight of the network and ***σ***_***w***_ is the standard deviation of the weight. Therefore, $\mu =\frac{\langle w\rangle }{\sigma_{w}}$.

At time ***t***, the number of these three groups is ***S***(***t***), ***I*(*t*)** and ***R*(*t*)**. From the network ***G*** = (***V*, *E*, *W***) topology parameters 〈***w***〉 = **0.119**, ***σ***_***w***_ = **0.519**, so ***μ*** = **0.22**, $\lambda =f(t)=e^{-0.22(t-t_{0})}$. Finally, all nodes except for the opinion leader node have recovery behavior. The recovery probability depends on the degree of network information flow, the collaboration needs of nodes, and the community’s investment in node recovery resources. Therefore, to facilitate the calculation, the overall recovery probability is set as ***γ*** = **0.1.**

The SIR model equations of opinion leaders are:

$$\left\{ \begin{array}{l} \frac{dI(t)}{dt}=e^{-0.22(t-t_{0})}I(t)S(t)-0.1I(t)\\ \frac{dS(t)}{dt}=-e^{-0.22(t-t_{0})}I(t)S(t)\\ \frac{dR(t)}{dt}=0.1I(t) \end{array}\right.$$ (5)

### Robustness analysis method

#### Description of failure process

In the multi-project KCN, our previous research [52] divided nodes and designed failure modes based on the number of users participating in projects. However, according to node attribute analysis, the node that participated in the most projects will not necessarily be the node with the highest out-strength or betweenness. Further, the strength and betweenness of nodes with the same number of participating projects will also be very different. Therefore, the nodes are divided into knowledge contribution nodes, knowledge dissemination nodes, opinion leader nodes and SHNs according to their structural attributes. Further, the impact of the behavior failure of these different node types on the performance of the multi-project KCN are analyzed.

According to the node attribute analysis, high intensity actively participate in knowledge collaboration (i.e., they actively contribute knowledge), and high-medium nodes are important "bridge" nodes that maintain the efficiency of knowledge collaboration in the network (i.e., they actively spread knowledge). In addition, the Local Motors OSC data shows that some nodes in the multi-project network have professional knowledge and are active in leading the flow of information and knowledge collaboration. They are at the core of the network and are the focus users that most nodes follow. These opinion leader nodes have typical authority and influence: they (1) have professional knowledge and actively contribute knowledge in the community, (2) are the center of information and communication hub in the community, and (3) are the most cohesive members who can influence the behavior and willingness of other members.

In the multi-project network, users select which projects they wish to participate in (i.e., through collaboration and discussion), often forming groups that are bound by particular projects. Users (i.e., nodes) in the structural hole position fulfill the intermediary function of connecting different complementary resources and knowledge groups. Further, they can attain the advantages of controlling the inside and outside of the group [31], allowing them to better understand the different interests in the community and provide guidance for community managers to effectively formulate policies. In addition to their location advantages, SHNs also have an important impact on innovation and individual or organizational behavior. Therefore, we can effectively analyze the impact of the collaborative behavior failure of SHNs on network robustness, providing an effective basis for the community to formulate management strategies.

In summary, the above content presents the failure modes of collaboration behavior for the different network nodes (i.e., opinion leaders, knowledge contributors, knowledge disseminators, and SHNs) and analyzes the impacts of these respective failures on the robustness of the OSC. In the multi-project network, the failure process of collaboration behavior is simulated by reducing edge weights. The respective descriptions and calculation processes of the four edge failure modes are shown in Table 6.

**Fig 3:**
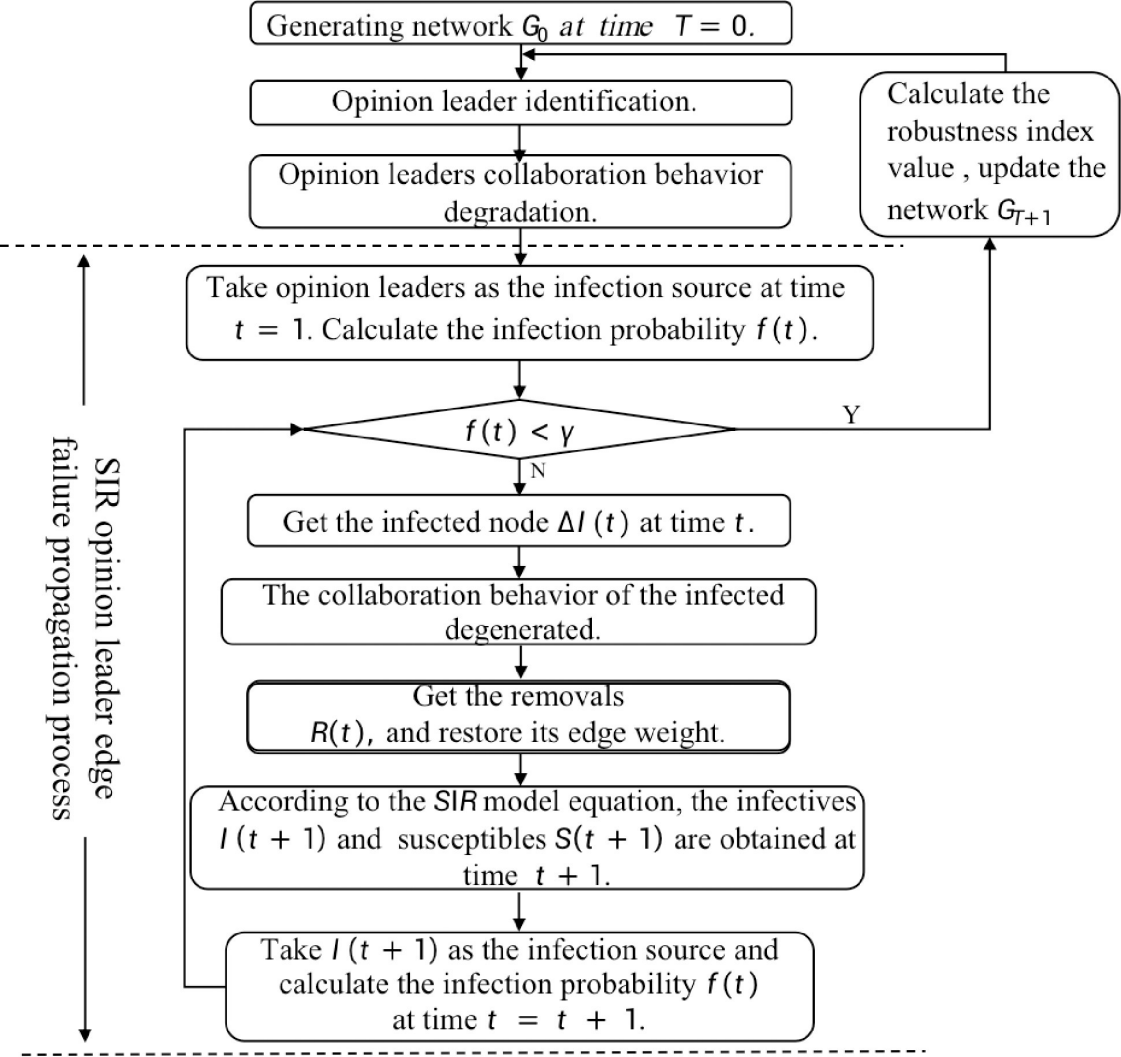
The simulation process of edge failure of opinion leader nodes.

**Table 6:** Edge failure modes.

	Failure Mode Description	Failure Simulation Calculation Process
Edge failure mode	edge failure of opinion leader nodes(CN)	(1) Reduce the directed edge weight of the first identified opinion leader node to the original value ε times. (2) Taking the opinion leader node as the initial infection source into the SIR model, reduce the weight of the infected node to the original value ε times. At the end of the evolution, calculate the robustness index value. Take this as the current network, then identify the opinion leader node and repeat steps (1) and (2). Repeat n times to simulate the most influential node and its behavior failure propagation mode. This simulation process is shown in Fig 3.
	edge failure of knowledge contribution nodes (WN)	Sort the nodes generated by the network according to their out-strengths. Based on the sorting result, select the first node to reduce the weight of the directed edge to the original value ε times. Take this as the current network, then calculate the out-strengths of nodes and sort to reduce the directed edge weight of the top node. Repeat n times to simulate the edge failure of knowledge collaboration nodes.
	edge failure of knowledge dissemination nodes (BN)	Sort the nodes generated by the network according to their node betweennesses. Based on the sorting result, select the first node to reduce the weight of the directed edge to the original value ε times. Take this as the current network, then calculate the node betweennesses of nodes and sort to reduce the directed edge weight of the top node. Repeat n times to simulate the edge failure of knowledge betweenness nodes.
	edge failure of SHNs(SN)	Sort the nodes generated by the network according to their hierarchy in the structural hole index. Based on the sorting result, select the first node to reduce the weight of the directed edge to the original value ε times. Take this as the current network, then calculate the hierarchy of nodes and sort to reduce the directed edge weight of the top node. Repeat *n* times to simulate the edge failure of SHNs.
Random failure	edge failure of random nodes (RN)	Randomly select a node to reduce the weight of the directed edge to the original value ε times. Repeat n times to simulate the edge failure of random nodes.

#### Robustness evaluation index

Network robustness can be defined as the degree of retention of network performance when network nodes or edges fail [64]. The impact of such failure for the KCN of an OSC includes (1) the destruction of network connectivity, which reduces the knowledge collaboration intensity, and (2) the decrease of network efficiency, which increases the difficulty of knowledge collaboration. As such, the robustness evaluation index proposed in this paper includes both network connectivity and weighted efficiency.

*Relative size of network connectivity*, ***S***. To reflect the degree of network connectivity retention after the network is attacked, the relative network connectivity size, ***S***, is defined as the relative size of the largest connected sub-graph node intensity of the network:

$$S=\frac{S_{lc}^{\prime }}{S_{lc}}$$ (6)

where $S_{lc}^{\prime }$ is the sum of the node intensity of the maximum connected sub-graph of the network after being attacked, and ***S***_***lc***_ is the sum of the node intensity of the original network. The calculation formula for the sum of the node intensity is

$$S_{lc}=\sum_{j=1}^{N}w_{ij}$$ (7)

where ***N*** is the total number of nodes in the network and ***w***_***ij***_ is the edge weight of nodes ***i*** and ***j***. The smaller the value of ***S***, the greater the decrease in knowledge collaboration intensity after the network is attacked (i.e., the lower the robustness of connectivity), and vice versa.

*Relative size of knowledge collaboration efficiency*, *H*. Network efficiency describes the difficulty of information dissemination. It is expressed as the sum of the efficiency of all nodes, where node efficiency is the reciprocal of the shortest path between two nodes [65]. In the directed weighted network, the efficiency of knowledge collaboration, ***E***_***G***_, is expressed as:

$$E_{G}=\frac{1}{n(n-1)}\sum_{i\neq j}\frac{1}{{\left(d_{w}\right)}_{i,j}}$$ (8)

where the directed weighted shortest path, (***d***_***w***_)_***i*,*j***_, is the minimum sum of the weights necessary to travel from nodes ***i*** to ***j***. To reflect the degree of knowledge collaboration efficiency retention after the network is attacked, the relative size of knowledge collaboration efficiency size, ***H***, is defined as

$$H=\frac{{E_{G}}^{\prime }}{E_{G}}$$ (9)

where ***E***_***G***_′ is the weighted efficiency of the attacked network and ***E***_***G***_ is the weighted efficiency of the original network. The value range of ***H*** is [**0,1**]. When ***H*** = **0**, network efficiency drops to its lowest after the attack, that is, designers in the network do not have any form of collaboration. When ***H*** = **1**, the efficiency of the whole network remains at the original level, where the failure of edge weights has no impact on network efficiency.

## Section 4 Results

### Robustness simulation test and result analysis

Based on the construction of a semantic-based KCN and the propagation effect of opinion leaders, Python 3.7 programming is used to simulate the change trend of the index value after the network faces the failure of the collaborative behavior of different nodes. Origin-pro 9.0 software is used to create the comparison chart of the experimental results.

**Fig 4:**
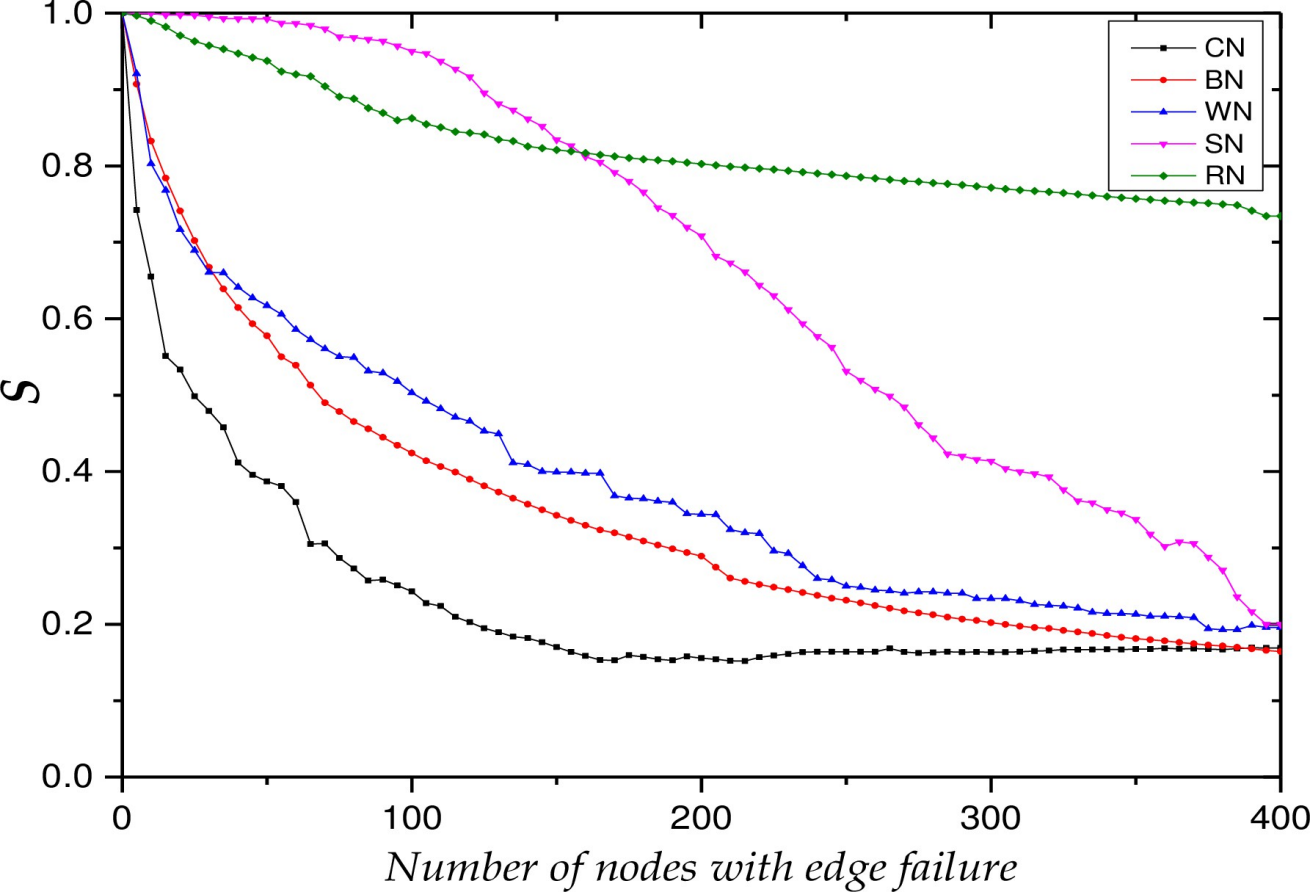
The trends of the relative size of network connectivity, *S*.

**Fig 5:**
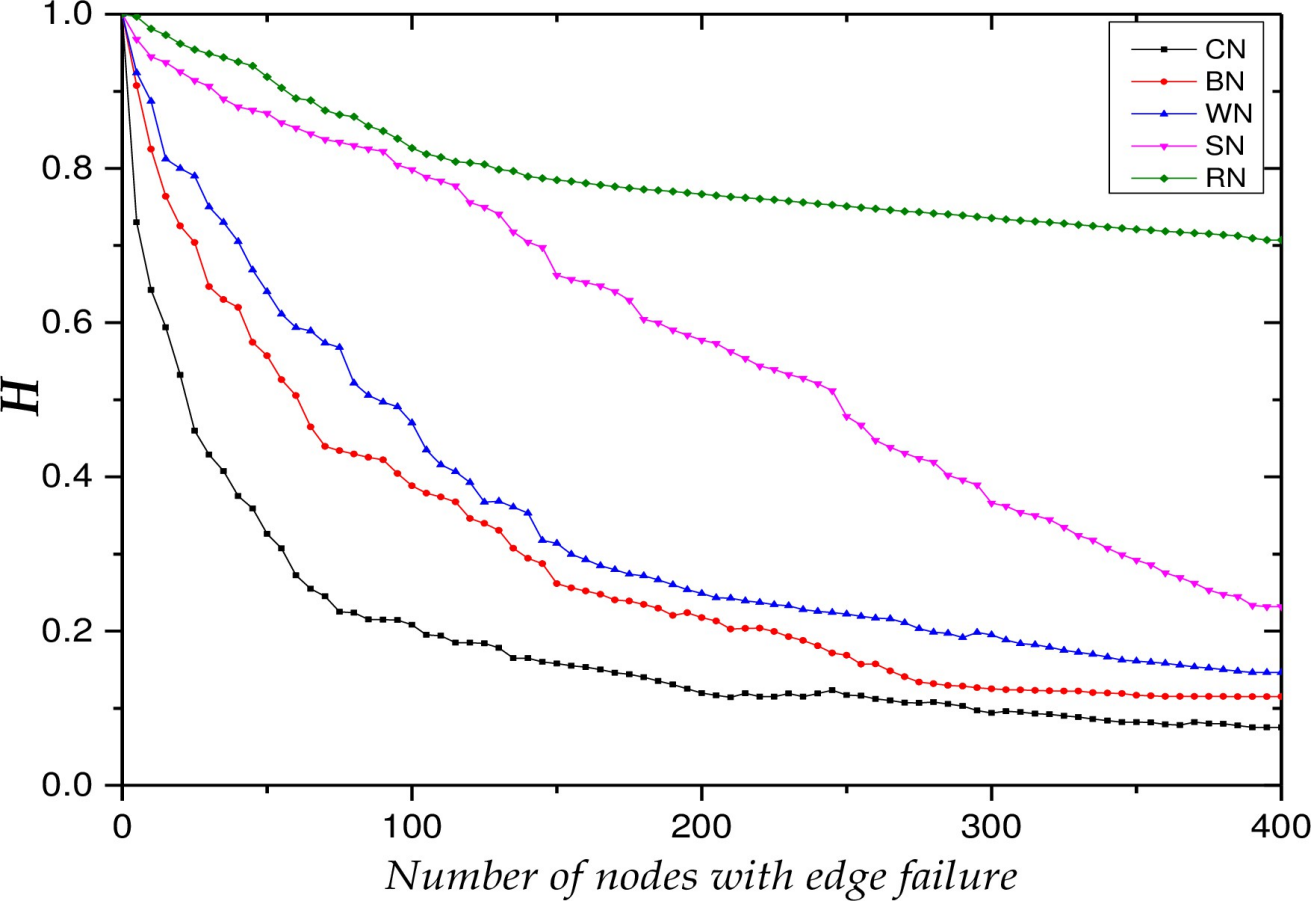
The trends of the relative size of weighted efficiency, H.

The test starts from *T* = 0 to simulate the change of the network robustness index during the continuous decline of collaborative behavior. To clearly observe the influence of knowledge collaboration behavior degradation on network robustness, the failure coefficient is taken as ***ε*** = **0.8**. In the propagation process of the failure of opinion leaders’ collaborative behavior, the initial time is set at ***t***_**0**_ = 0. Figs 4 and 5 show the trends of the relative size of network connectivity and knowledge collaboration efficiency, respectively, under the five edge failure modes described in Table 6.

Figs 4 and 5 show that the multi-project KCN shows the characteristics of robustness and fragility when facing edge failures: they are robust under random failures and fragile under deliberate failures. This is because the node strength of the network satisfies a power-law distribution, where a small proportion of nodes have a large number of collaborative relationships, while the majority of nodes engage only in a small number of collaborative relationships. Moreover, nodes with high strength have large edge weights, while nodes with low strength have relatively small edge weights. As most edge weights are small, when random failures occur, the damage to the overall network is minimal. Therefore, the index value decreases least during the edge failure of random nodes (RN), followed by SHNs (SN), knowledge contribution nodes (WN), knowledge dissemination nodes (BN) and opinion leader nodes (CN).

A paired T-test is conducted on the index value trend data under the five failure modes collected from the simulation experiment, as shown in Table 7. The test results show that the index values under different failure modes are significantly different. Based on the above analysis, it can be concluded that regarding network robustness following edge failure: RN>SN>WN>BN>CN.

**Table 7:** Paired sample T-test under different edge failure modes.

	Failure mode	M	SD	95% Confidence		t	df	Sig
				Lower limits	Upper limits			
	RN-SN	0.168229	0.015822	0.13665	0.19980	10.632	68	0.000
	SN-WN	0.254688	0.014057	0.22625	0.28312	18.118	39	0.000
H	WN-BN	-0.02577	0.006295	-0.0385	-0.0130	-4.094	39	0.000
	BN-CN	0.228375	0.014701	0.19780	0.25894	15.534	21	0.000
	RN-SN	0.125403	0.021894	0.08177	0.16902	5.728	74	0.000
	SN-WN	0.36912	0.013490	0.34196	0.39627	27.361	46	0.000
S	WN-BN	0.028124	0.003065	0.02195	0.03429	9.176	46	0.000
	BN-CN	0.144010	0.010907	0.12088	0.16713	13.203	16	0.000

Further, Fig 4 shows that the edge failures of SHNs do not initially affect the relative size of network connectivity. It is only when the number of SHNs with edge failures reaches 15 that the relative size of network connectivity slowly decreases, and when the number reaches 100, it then quickly decreases to its lowest value. In contrast, Fig 5 shows that the efficiency of knowledge collaboration maintains a relatively stable decline rate during SHN edge failure, which indicates that nodes with strong structural holes are distributed on critical paths in the network rather than nodes with high strength values.

The respective decline trends (shown in Figs 4 and 5) for other edge failure modes are relatively consistent, that is, they have an initial rapid decline followed by a slow decline until the lowest value is reached. Due to the following effect of opinion leaders on other users, the index value of the network drops to 50% of the original value after the edge failure of the 28th opinion leader node, and the value of the relative size of network connectivity reaches its lowest point after the edge failure of the 160th opinion leader node. During the network’s evolution, nodes continuously restore their willingness to collaborate, resulting in an increase in network connectivity. However, the relative size of network connectivity has only recovered by 3% and the relative size of knowledge collaboration efficiency has hardly changed. This is because the proportion of marginal nodes in the multi-project KCN is very high, and the heterogeneity of these nodes is not obvious. The immune nodes are basically these nodes, so immunization has almost no effect on network performance recovery. The propagation influence of opinion leader nodes is relatively low in a multi-project KCN compared to a single-project KCN. This is mainly due to: (1) the large scale of the multi-project KCN and the looser network, which results in a very limited dissemination depth and breadth; and (2) nodes are aggregated on a project basis, which results in relatively more difficult propagation across projects.

Finally, the respective index value trends of the edge failures of the knowledge contribution nodes and knowledge dissemination nodes are relatively consistent, and their respective ranges of decline are not significantly different. This is particularly true in the early stages of the simulation experiment: the trends of knowledge contribution node failure and knowledge dissemination node failure overlap. With the continuous evolution of the network, the index value becomes more sensitive to the edge failure of knowledge contribution nodes. This is the opposite conclusion to the edge failure of knowledge contribution nodes in the single-project KCN, indicating that larger networks are looser, so the propagation behavior is more important for maintaining network connectivity and collaboration efficiency.

### Conclusion

This paper analyzes the characteristics of the OSC-based multi-project KCN, uses structural hole theory to identify key nodes, designs edge failure modes for different nodes and conducts robustness research. The analysis results show that the KCN has the lowest robustness when facing the edge failure of opinion leader nodes, followed by knowledge dissemination nodes, knowledge contribution nodes, SHNs and random nodes. The edge failure of opinion leader nodes causes the lowest network robustness because of the propagation effect of these nodes. Further, SHN failure has only a small initial impact on connectivity, while knowledge collaboration efficiency decreases rapidly. This indicates that the edge failure of SHNs can leave the network in a state of high connectivity and low efficiency, but network connectivity will continue to decline as more edge failures occur. Based on the experimental results, the following management suggestions are proposed:

1. OSCs should effectively identify opinion leader nodes and give them more attention and protection to reduce the risk of them losing their willingness to collaborate. Further, in the daily community operation process, attention should be paid to the opinions of these nodes regarding community management, project setup, personnel coordination and other aspects. OSCs should establish competitive incentive mechanisms to enable opinion leaders to participate in more project collaboration and improve the quality and intensity of knowledge collaboration. In community ecological construction, community managers should also focus on improving the timely release of information and the updating of basic information on projects, task modules and personnel introductions, to avoid other users blindly following opinion leaders. OSCs should also continuously attract more users to join them. When user behavior fails, resources should first be concentrated on restoring the collaborative behavior of core nodes to reduce a rapid decline in network performance.
2. The collaborative behavior of knowledge dissemination nodes and knowledge contribution nodes is very important for maintaining network performance, particularly in protecting knowledge dissemination nodes that occupy key positions in the network. Managers should effectively identify these two types of nodes, develop corresponding incentive mechanisms for their core demands, strengthen the promotion of the importance of knowledge dissemination, and improve the awareness and willingness of users to disseminate knowledge.
3. The persistent failure of the collaborative behavior of SHNs can also lead to a significant decline in network performance. Such nodes are a breakthrough for enterprise innovation and improving competitive advantage. Therefore, managers should pay more attention to SHNs, encouraging them to actively participate in the collaboration of multiple projects.

The following abbreviations are used in this manuscript:

## Supporting information

S1 Data (XLSX)

S2 Data (XLSX)

S3 Data (XLSX)

S4 Data (XLSX)

S5 Data (XLSX)

S6 Data (XLSX)

S7 Data (XLSX)

S8 Data (XLSX)

S9 Data (XLSX)

S10 Data (XLSX)

S11 Data (XLSX)

## Abbreviations

KCN: Knowledge Collaborative Network
OSC: Open source communitiy
OSP: Open source project
SHN: structural hole nodes
CN: edge failure of opinion leader nodes
WN: edge failure of knowledge contribution nodes
BN: edge failure of knowledge dissemination nodes
SN: edge failure of SHNs
RN: edge failure of random nodes

## References

[1] Shaikh Maha, Levina Natalia. Selecting an open innovation community as an alliance partner: Looking for healthy communities and ecosystems.Research Policy: A Journal Devoted to Research Policy, Research Management and Planning 2019, 48(8). doi: 10.1016/j.respol.2019.03.011

[2] Global Open Source Ecology Research Report (2022). http://www.caict.ac.cn/kxyj/qwfb/bps/202209/t20220916_409031.htm.

[3] Sanchez H.; Oliveira D.; Shands D. Leveraging Team Dynamics to Predict Open-source Software Projects’ Susceptibility to Social Engineering Attacks[J]. arXiv 2021, arXiv: 2106. 16067.

[4] Chen G. P.; Wei J.; Li T. Y. Open source community: research context,knowledge framework and research prospects[J]. Foreign Economics & Management 2021, 43(02): 84–102. doi: 10.16538/j.cnki.fem.20200904.402

[5] Li Y. Z.; Zhang S.; Zhang X. D. A review of key impacting factors in the evolution process of open source design. Science Research Management 2020, 41(08): 13–22. doi: 10.19571/j.cnki.1000-2995.2020.08.002

[6] Crowston K.; Annabi H.; Howison J. Defining open Source software project success[C]//Proceedings of International Conference on Information Systems 2003. USA: Seattle WA, 2003:327–340.

[7] Griffith T. L.; Sawyer R. E. Multilevel knowledge and team performance. Journal of Organizational Behavior 2010, 31(7):1003–1031.

[8] Singh P. V.; Tan Y.; Mookerjee V. Network Effects: The Influence of Structural Social Capital on Open Source Project Success. MIS Quarterly 2011, 35(4):813–829.

[9] Ransbotham S.; Kane G. C. Membership Turnover and Collaboration Success in Online Communities: Explaining Rises and Falls from Grace in Wikipedia. MIS Quarterly 2011, 35 (3):613–627.

[10] Qin M.; Qiao H.; Chen L. H. Online yser contribution behavior in enterprise -hosted open innovation communities based on complex adaptive system: An example of chinese famous enterprise-hosted community. Management Review 2015, 27(01): 126–137. doi: 10.14120/j.cnki.cn11-5057/f.2015.01.012

[11] Li Y.Z.; Zhang S.; Zhang X. D. Impact of Online Evaluation on Members′Innovation Performance in Open Source Design Community——A Case Study for Local Motors. Journal of Management Science 2020, 33(03): 52–62.

[12] Lü L. Y.; Lu J. A, Zhang Z. K. Complex System and Complex Science 2010.

[13] Iyer S.; Killingback T.; Sundaram B. Attack robustness and centrality of complex networks. PloS One 2013, 8(4):e59613. doi: 10.1371/journal.pone.0059613 23565156 PMC3615130

[14] Budak C.; Agrawal D.; Abbadi A. E. 2011 Proceedings of the International World Wide Web Conference Committee Hyderabad, India, March 28-April 1, 2011: 665.

[15] Bonacich P. Factoring and weighting approaches to status scores and clique identifification. Journal of Mathematical Sociology 1972, 2, 113–120. doi: 10.1080/0022250X.1972.9989806

[16] Zhu Z.; Cui Z.; Ding X.; Rui M. Identifying opinion leaders in major sudden public opinion spread based on entropy-weighted grey correlation model. Journal of China Society for Scientific Technical Information. 2017, 36, 706–714.

[17] Han Z. M.; Chen Y.; Liu W. Research on note influence analysis in social networks. Journal of Software 2017, 28(1): 84–104.

[18] Yu H.; Liu Z.; Li Y. Key nodes in complex networks identified by multi-attribute decision-making method. Acta Physica Sinica 2013, 62, 54–62, CNKI:SUN:WLXB.0.2013-02-008.

[19] Han Z. M.; Wu Y.; Tan X. S. Ranking key nodes in complex networks by considering structural holes. Acta Physica. Sinica.2015, 5(64): 058902. doi: 10.7498/aps.64.058902

[20] Zhou H.L. Research on Dynamic Robustness of Knowledge Collaboration Network of Open Source Product Community. Ph.D. Thesis, University of Science and Technology Beijing, Beijing, China, 2018.

[21] Zhou H, Zhang X, Hu Y. Robustness of open source product innovation community’s knowledge collaboration network under the dynamic environment. Physica A: Statistical Mechanics and its Applications 2020, 540, 122888, doi: 10.1016/j.physa.2019.122888

[22] Xu B. C.; Zhang X. D. Opinion leader identification and following effect simulation in the open source community. Information studies: Theory & Application 2019, 42, 101–107, doi: 10.16353/j cnki.1000-7490.2019.12.016.

[23] Lei S.; Zhang X.; Liu S. Dynamic Robustness of Open-Source Project Knowledge Collaboration Network Based on Opinion Leader Identification[J]. Entropy 2021, 23(9): 1235, doi: 10.3390/e23091235 34573860 PMC8470236

[24] Burt R. S.; Reinforced structural holes. Social Networks 2015, 43: 149–161.

[25] Yan L. Y.; Zhang Y. Analysis of brokerage roles of online academic social users based on structural hole theory. Journal of intelligence 2022. 41(11): 164–170.

[26] Yan Y. L.; Zhang Y.; Cha X. J. Research progress of structural holes theory application abroad. Knowledge, Learning & Management 2019, 190(4): 104–112. doi: 10.13366/j.dik.2019.04.104

[27] Cai Y. H.; Fu L. F.; Liang J. Evolution of Alliance Relationship, Network Structure Hole and Firm’s Cooperative Innovation Performance. Forum on Science and Technology in China 2021, 000(010):94–103. doi: 10.13580/j.cnki.fstc.2021.10.011

[28] He X. J.; Wu S. S.; Wu Y. Y.; Pang T. Identification of Structural Hole Spanners and its Role Evolution in Patent Transfer Networks: An Empirical Study on the China Greater Bay Area. Science of Science and Management of S.&.T 2022,43(4):75–94, doi: 10.3969/j.jssn.1002–0241.2022.04.75.20.

[29] Wilson T. D. Human information behavior. Informing Science the International. Journal of An Emerging Transdiscipline 2000, 3(2), 49–56. doi: 10.1002/sdr.4260070210

[30] Ballinger G. A.; Cross R., & Holtom B. C. The right friends in the right places: understanding network structure as a predictor of voluntary turnover. Journal of Applied Psychology 2016, 101(4), 535–548. doi: 10.1037/apl0000061 26595755

[31] Hu Y.; Zhou H,L. Open source design network’s micro features: An analysis based on the structural holes theory. Science Technology and Engineering 2018, 18 (12): 235–241.

[32] Burt R. S. Structural Holes and Good Idea. American Journal of Sociology 2004.110(2): 349–399.

[33] Kim Y. K.; Lee D. J.; Ji H. Influential users in social network services: the contingent value of connecting user status and brokerage. The data base for advances in information systems 2018, 49(1):13–31.

[34] Zhou J.; Shin S. J.; Brass D. J.; Choi J.; Zhang Z. X. Social networks, personal values, and creativity: evidence for curvilinear and interaction effects. Journal of Applied Psychology 2009, 94(6), 1544–1552. doi: 10.1037/a0016285 19916661

[35] Wang C.; Rodan S.; Fruin M.; Xiaoyan X. U. Knowledge networks, collaboration networks, and exploratory innovation. Academy of management journal 2014, 57(2):484–514.

[36] Stea D.; Pedersen T. Not all brokers are alike: creative implications of brokering networks in different work functions. Human Relations 2017, 70(1):668–693. doi: 10.1177/1/0018726716672921

[37] Figueiredo C.; Chen W.; Azevedo J. Central nodes and surprise in content selection in social networks. Computers in human behavior 2015, 51: 382–392. doi: 10.1016/j.chb.2015.04.070

[38] Wang W.; Meng T. Customer Innovation Behavior in Brand Community: Effects of Centrality and Structure Hole. Journal of Management Science 2021, 34(3): 135–147. doi: 10.3969/j.jssn.1672-0334.2021.03.011

[39] Goyal S.; Vega-Redondo F. Structural holes in social networks. Journal of Economic Theory 2007, 137(1): 460–492.

[40] Ren Y.; Chen J.; Riedl J. The Impact and Evolution of Group Diversity in Online Open Collaboration. Management Science 2016, 62(6):1668–1686.

[41] Huang H. Y.; Qize. Analysis of the Structure and Evolution of an Open-Source Community. Journal of Computing & Information Science in Engineering 2011.

[42] Guo S. Z.; Zhe M. L.; Chen Z. Strength-Strength and Strength-Degree Correlation Measures for Directed Weighted Complex Network Analysis. IEICE Transactions on Information and Systems, 2011, 94(11):2284–2287.

[43] Barrat A.; Barthélemy M.; Vespignani A. Weighted evolving networks: coupling topology and weights dynamics. Physical Review Letters, 2004, 92(22):228701. 15245264 10.1103/PhysRevLett.92.228701

[44] Paul G.; Tanizawa T.; Havlin S.; Stanley H. E. Optimization of robustness of complex networks. European Physical Journal B, 2005, 48(1), 149–154.

[45] Bellingeri M.; Cassi D. Robustness of weighted networks. Physica A: Statistical Mechanics & Its Applications 2018, 489,47–55, doi: 10.1016/j.physa.2017.07.020

[46] Albert R. H.; Jeong, Barabási, A. Error and attack tolerance of complex networks. Nature 2000. 406(6794): 378–382. 10935628 10.1038/35019019

[47] Fuge M.; Tee K.; Agogino A.; Maton N. Analysis of Collaborative Design Networks: A Case Study of OpenIDEO. Journal of Computing & Information Science in Engineering 2014, 14, 226–235, doi: 10.1115/1.4026510

[48] Zhang X.; Zhou H.; Hu Y. Dynamic robustness of knowledge collaborative network under mass collaboration environment. Computer Integrated Manufacturing Systems, CIMS 2017, 23, 2353–2360, doi: 10.1016/j.physa.2017.08.092

[49] Zhou H. L.; Zhang X. D. Dynamic robustness of knowledge collaboration network of open source product development community. Physica A: Statistical Mechanics & Its Applications 2018, (490):601–612. doi: 10.1016/j.physa.2017.08.092

[50] Lei S.; Zhang X.; Xie S.; Zheng X. Dynamic robustness of semantic-based collaborative knowledge network of open source project. Entropy 2021, 23, 391, doi: 10.3390/e23040391 33806184 PMC8066244

[51] Lei S. J. Robustness of Open-Source Community Knowledge Collaboration Network based on multiple failure modes. Ph.D. Thesis, University of Science and Technology Beijing, Beijing, China, 2022.

[52] Zhang X.; Lei S.J; Sun J. Kou W. Robustness of Multi-Project Knowledge Collaboration Network in Open Source Community. Entropy 2023; 25(1):108. doi: 10.3390/e25010108 36673249 PMC9857583

[53] Davis G.F.; Yoo M.; Baker W.E. The Small World of the American Corporate Elite, 1982–2001. Acoustics, Speech, and Signal Processing Newsletter, IEEE 2003, 1, 51–57, doi: 10.1177/14761270030013002

[54] Bellingeri M. Cassi D. Vincenzi S. Efficiency of attack strategies on complex model and real-world networks[J].Physica A: Statistical Mechanics & Its Applications 2014(414): 174–180. doi: org/10.1016/j.physa.2014.06.079

[55] Ahmad W.; Hasan O.; Pervez U.; Reliability modeling and analysis of communication networks [J]. Journal of Network and Computer Applications 2017, 78: 191–215.

[56] Ba Z.; Li G.; Zhu S. Knowledge diffusion mechanism of scientific cooperation network [J]. Journal of Library Science in China 2016, 42 (5): 68–84.

[57] Li C.; Jiang G.; Song Y. Epidemic spreading in dynamic small world networks with community structure [J]. Complex Systems and Complexity Science 2014, 11 (3): 33–39.

[58] Liu Y.; Peng L.; Zhao Z. The evolution of rumor spread on micrblog based on small world network [J]. Complex Systems and Complexity Science 2014, 11 (4): 54–60.

[59] He J.; Liu J.; Xu F. et al. Modeling and simulating of propagation dynamics of time banditry behaviour based on SIR model [J]. Application Research of Computers 2018, 35 (5): 1 360–1 364.

[60] Lazarsfeld P. F. Public opinion and the classical tradition. Public Opinion Quarterly 1957, 21(1), 39–53.

[61] Katz E. The two-step flow of communication: An up-to-date report on a hypothesis. Public Opinion Quarterly 1957, 21(1), 61–78.

[62] Burt R. S. Structural holes: the social structure of competition[M]. Campetition, MA: Harvard University Press, 1992.

[63] Burt R. S. The social capital of opinion leaders. Annals of the American academy of political and social science 1999, 556:37–54.

[64] Du W.; Cai M.; Du H. F. Study on indices of network structure robustness and their application[J]. Journal of Xi’an Jiaotong University 2010, 44(04): 93–97.

[65] Wang J.; Wu X.; Chen Y. Invulnerability simulation of weighted complex networks with different information. Journal of Central South University(Science and Technology) 2013, 44, 1888–1893, CNKI:SUN:ZNGD.0.2013-05-023.

